# A monocentric, randomized, double-blind, controlled crossover trial of nasturtium (*Tropaeolum majus*) on the lipid regulator prostaglandin E_2_

**DOI:** 10.3389/fnut.2023.1223158

**Published:** 2023-08-03

**Authors:** Corinna Herz, Linda Frei, Hoai T. T. Tran, Sophie Claßen, Jenny Spöttel, Mareike Krell, Franziska S. Hanschen, Marjan Arvandi, Nadine Binder, Monika Schreiner, Sascha Rohn, Evelyn Lamy

**Affiliations:** ^1^Molecular Preventive Medicine, University Medical Center and Faculty of Medicine, University of Freiburg, Freiburg, Germany; ^2^Institute of Food Chemistry, Hamburg School of Food Science, University of Hamburg, Hamburg, Germany; ^3^Plant Quality and Food Security, Leibniz Institute of Vegetable and Ornamental Crops, Großbeeren, Germany; ^4^Department of Public Health, Health Services Research and Health Technology Assessment, Institute of Public Health, Medical Decision Making and HTA, UMIT TIROL, University for Health Sciences and Health Technology, Hall in Tirol, Austria; ^5^Institute of General Practice/Family Medicine, Faculty of Medicine and Medical Center, University of Freiburg, Freiburg, Germany; ^6^Institute of Food Technology and Food Chemistry, Technische Universität Berlin, Berlin, Germany

**Keywords:** *Tropaeolum majus*, benzyl isothiocyanate, Brassicaceae, nutrition-based intervention, personalized nutrition, responder analysis

## Abstract

**Scope:**

As prostaglandin E2 (PGE_2_) has important roles in physiological and inflammatory functions, a double-blind randomized controlled crossover study to investigate the potential of nasturtium (*Tropaeolum majus*) for modulating PGE_2_ was conducted, aiming at clarifying the role of benzyl isothiocyanate (BITC). As secondary parameters leukotriene 4 (LTB_4_), and cytokine release (tumor necrosis factor alpha, TNF-α; interleukins IL-1β, IL-10, and IL-12) were quantified.

**Methods and results:**

Thirty-four healthy female participants consumed 1.5 g nasturtium containing BITC, (*verum*) or no BITC (control) twice a day for 2 weeks each. Nasturtium intervention resulted in an increase in mean PGE_2_ levels in serum samples (*verum*: 1.76-fold, *p* ≤ 0.05; control: 1.78-fold, *p* ≤ 0.01), and *ex vivo* stimulated peripheral blood mononuclear cells (PBMC) (*verum*: 1.71-fold, *p* ≤ 0.01; control: 1.43-fold). Using a pre-to-post responder analysis approach, 18 of 34 subjects showed *a* > 25% PGE_2_ increase in serum, while it was >25% decreased for 9 subjects (stimulated PBMC: 14 and 8 of 28, respectively). Under the selected conditions, the BITC content of nasturtium did not affect the observed changes in PGE_2_. *Verum* intervention also increased mean LTB_4_ serum level (1.24-fold, *p* ≤ 0.01), but not in LPS stimulated PBMC, and significantly increased TNF-*α* release in stimulated PBMC after 3 h (*verum*: 1.65-fold, *p* = 0.0032; control: 1.22-fold, *p* = 0.7818). No change was seen in the anti-inflammatory cytokine IL-10, or the pro-inflammatory cytokines IL-1β, and IL-12.

**Conclusion:**

In contrast to the previously reported *in vitro* results, on average, LPS activated PBMC and serum from both groups showed increased PGE_2_ levels. Further analyses suggest that PGE_2_ release after intervention could possibly depend on the baseline PGE_2_ level. Identification of phenotypes that respond differently to the nasturtium intervention could be useful to establish personalized approaches for dosing phytopharmaceuticals medicines.

## 1. Introduction

Nasturtium (*Tropaeolum majus*, *T. majus*, Indian cress) from the Brassicales plant order is widely used in culinary and as dietary supplement. The plant is also a component in phytopharmaceuticals for the prevention and treatment of urinary tract infections, and against acute sinusitis or bronchitis (1, 2). Nasturtium contains large amounts of the prodrug glucosinolate (GLS) glucotropaeolin (benzyl GLS), especially in the leaves, flowers and seeds (3). This compound breaks down to benzyl isothiocyanate (BITC) by enzymatic cleavage by the plant’s endogenous myrosinase when the cell matrix is destroyed. There is good evidence for the pharmacological efficacy of BITC from *in vitro* studies (4–6). However, there is a lack of clear evidence on the role of BITC in the health-promoting or therapeutic efficacy of nasturtium in humans. Besides BITC, nasturtium is also rich in polyphenols such as anthocyanins, which have also been described as anti-inflammatory or antioxidant (7, 8). In a previous study, we demonstrated a concentration-dependent inhibition of the cyclooxygenase (COX)/prostaglandin E_2_ (PGE_2_) signaling pathway in bacterial lipopolysaccharide (LPS) stimulated peripheral blood mononuclear cells (PBMC) from healthy human volunteers upon treatment with a water extract of nasturtium (8). PGE_2_ is one of the most abundant prostanoids in the human body. It mediates pathogenic mechanisms, including the inflammatory response, in which it contributes to the development of the cardinal signs of acute inflammation. Inhibition of COX and subsequent reduction in the generation of prostanoids is the most established mechanism of action of non-steroidal anti-inflammatory drugs (9). However, nowadays PGE_2_ is also known to sustain important homeostatic functions and is considered important for the resolution of inflammation (10–12).

In our recent *in vitro* study, heat inactivation of myrosinase prior to extraction did not abolish the inhibitory effect on PGE_2_, thus questioning the relevance of BITC in these experiments (8). In contrast, in a 5-day human randomized, controlled trial conducted with the plant *Brassica carinata* from the order Brassicales, we found a significant reduction in serum PGE_2_ levels after consumption of a plant powder drink containing allyl isothiocyanate (AITC) and not after drinking the preparation devoid of AITC (13). Based on these findings, the present study aimed to clarify whether the basic research results shown *in vitro* could be transferred in the same way to the situation in humans, when consuming nasturtium. Thus, we conducted a monocentric, randomized, doubleblind, controlled crossover trial in order to determine the effect of regular nasturtium intake on PGE_2_ levels as primary parameter. By using nasturtium containing preformed BITC, and also nasturtium with inactivated myrosinase from which BITC could not be enzymatically released, we aimed to clarify the role of BITC in acting on PGE_2_. Secondary parameters included the quantification of leukotriene 4 (LTB_4_) levels, and also cytokine release (tumor necrosis factor alpha, TNF-α, interleukins IL-1β, IL-10, and IL-12) from *ex vivo* LPS stimulated PBMC from healthy human volunteers.

## 2. Materials and methods

### 2.1. Chemicals

Fetal calf serum (FCS), L-glutamine and phosphate buffered saline (PBS, without Ca and Mg), penicillin–streptomycin (P/S) solution, L-glutamine solution, RPMI-1640 and penicillin/streptomycin solution (10,000 U/mL and 10,000 μg/mL) were purchased from Gibco™, Life Technologies GmbH (Darmstadt, Germany). LPS from *Escherichia coli* O11:B4, standard BSA solution (10 mg/mL), ammonium persulfate, methanol (>99%) and Tween 20 were from Sigma Aldrich (Taufkirchen, Germany), LymphoPrepTM gradient was purchased from Progen Biotechnik GmbH (Heidelberg, Germany). Colistin sulfate salt was purchased from Thermo Fisher Scientific Inc. (Waltham, Massachusetts, USA). Trifluoracetic acid (TFA, >99%) was from AppliChem GmbH (Darmstadt, Germany). Pronase E (from *Streptomyces griseus*) was obtained from Merck KGaA (Darmstadt, Germany). Sodium hydroxide, formic acid (FA; 98%), acetonitrile (LC–MS grade), methylene chloride (GC Ultra Grade), and water Rotisolv HPLC were purchased from Carl Roth GmbH+Co. KG (Karlsruhe, Germany). C18ec solid phase extraction cartridges (3 mL, 200 mg) were obtained from Machery-Nagel GmbH & Co. KG (Düren, Germany). Benzyl cyanide (≥98%), benzonitrile (≥99.9%), and BITC (≥98.5%) were obtained from Sigma-Aldrich Chemie GmbH (Steinheim, Germany) and Na_2_SO_4_ anhydrous (≥99%) was from VWR International GmbH (Darmstadt, Germany). β-Mercaptoethanol was obtained from Merck KGaA (Darmstadt Germany). Ethylene diamine tetraacetic acid (EDTA, 99%, p.a.) and sodium dodecyl sulfate (SDS, ≥99%) was from Serva GmbH (Heidelberg, Germany).

### 2.2. Study population and ethical approval

**Figure 1:**
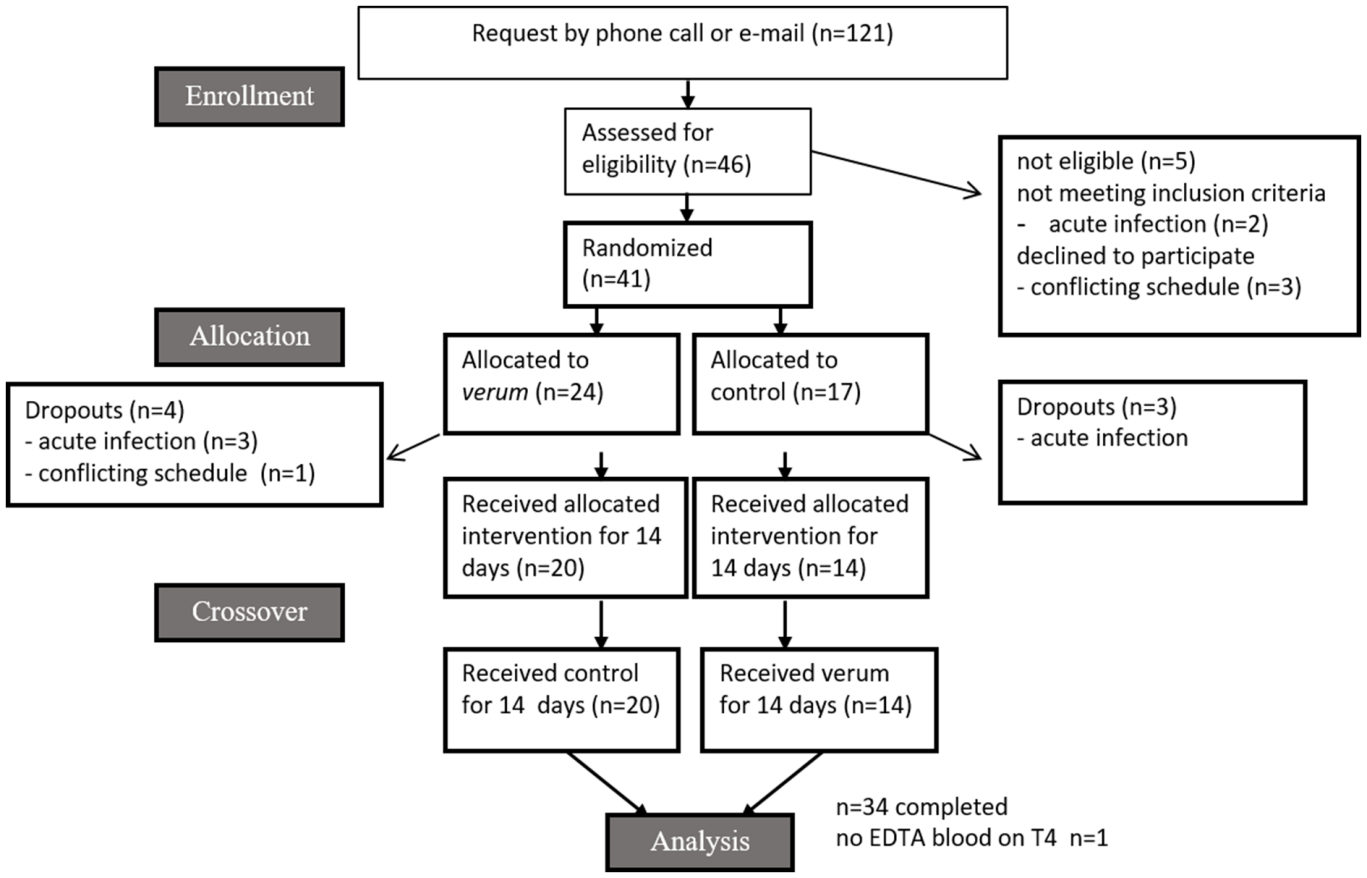
CONSORT flow diagram.

This study was conducted in accordance with the Declaration of Helsinki and approved by the Ethical Committee of the University of Freiburg (ethical vote number 322/18, 11/13/2018). This intervention trial was registered on German Clinical Trials Register (DRKS) with the ID: DRKS00016548. All participants provided written informed consent for study participation and received financial compensation approved by the ethics committee. A total of 46 healthy female subjects were recruited *via* posted flyers, social media, and personal communication in Freiburg, Germany. Subjects were screened for eligibility using a health questionnaire. As the immune system differs between men and women (14), the study was conducted with women only, so sex did not need to be considered as a confounding factor. The inclusion criteria for the study were as follows: age between 20 and 45 years, good health status and non-smoking. Exclusion criteria included: acute and chronic diseases, acute gastrointestinal ulcer, acute nephritis, pregnancy, breast feeding, intake of antibiotics (acute or during the last 3 months), severe allergies against vegetables of the order Brassicales and strong over- or underweight (BMI > 25 or < 18). Five subjects dropped out before randomization: two for health reasons (acute infection), and three had conflicting schedules with the planned study. During allocation, 7 participants dropped out: 6 due to acute illness and one due to conflicting schedules with the study. 34 participants completed the study. The flow diagram according to CONSORT guidelines for randomized trials is shown in Figure 1.

### 2.3. Nasturtium (*T. majus*) preparation

Standardized dried powder of *T. majus* flowers and leaves was provided by Repha GmbH (Langenhagen, Germany). A detailed phytochemical analysis of the plant powder was reported by us earlier (8). For consumption, every day subjects freshly prepared 1.5 g of plant powder plus a pinch of sugar (around 0.04 g), which was provided to them in a plastic medication cup with lid, by thoroughly suspending this mixture in 20 mL of drinking water and shaking for 20 s. Subsequently, the preparation was swallowed, with optional consumption of white bread. We used dried plant powder containing (1) the active enzyme myrosinase, which resulted in the formation of a defined amount BITC during the preparation step (*verum* preparation), and (2) containing heat-inactivated myrosinase, which did not produce ITC (control). In the *verum* 18.87 ± 4.5 μmol BITC and 2.33 ± 0.05 μmol benzyl nitrile (BCN) were detected, whereas the control contained 0.07 ± 0.06 μmol BITC and 4.22 ± 0.63 μmol BCN per serving. Prior to the intervention, aliquots of the plant powders were analyzed for the absence of relevant microbiological contamination following the guidelines of the *German Society for Hygiene and Microbiology* (DGHM, Münster, Germany) for dried products (herbs and spices) by Eurofins BioTesting Services Nord GmbH (Hamburg, Germany).

### 2.4. Study design and protocol

**Figure 2:**
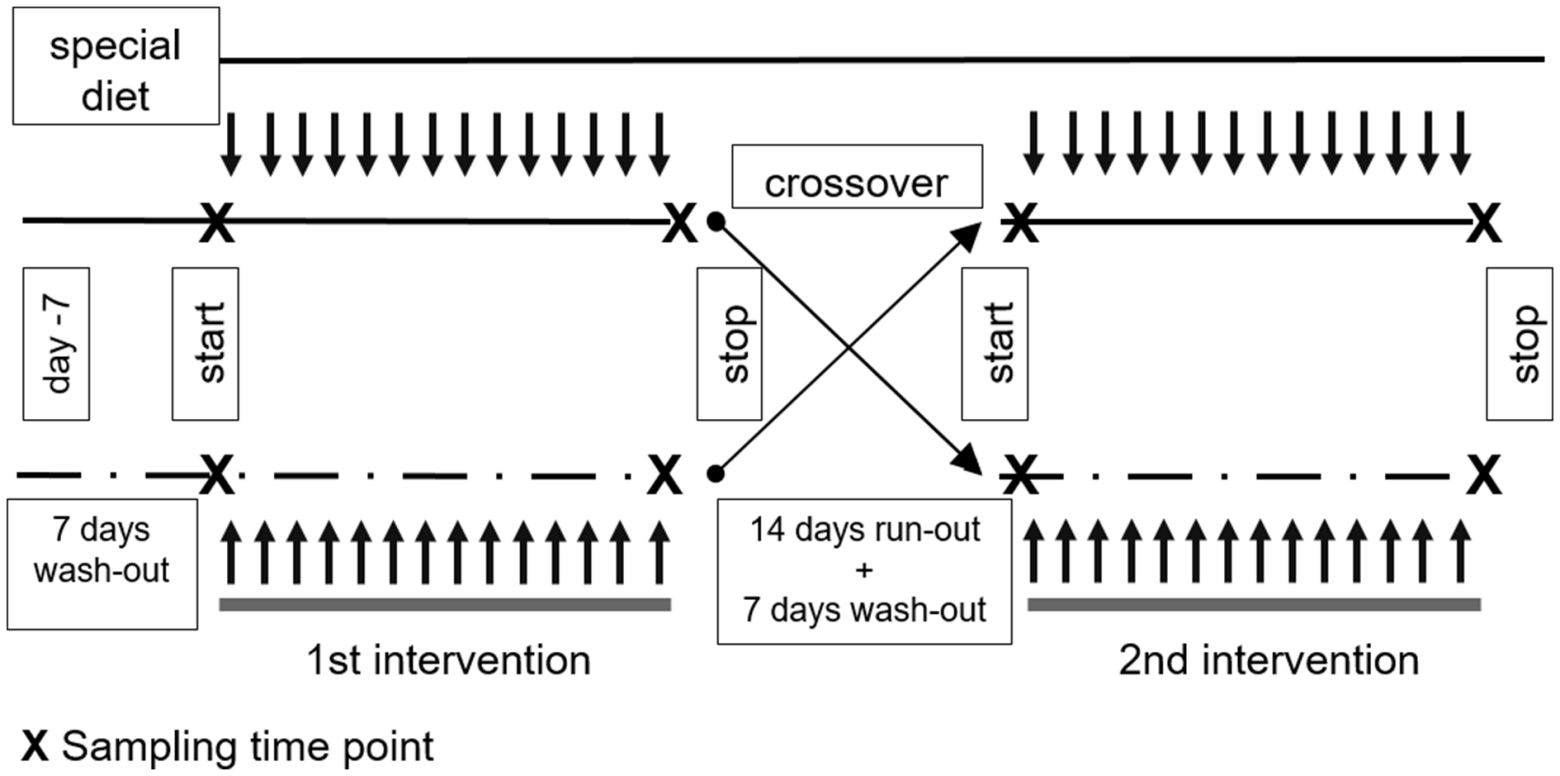
Study design, treatment and sampling time line. This study was conducted as a randomized, double-blind, controlled, crossover trial. Each subject participated in two 14-day intervention phases separated by a 14-day run-out phased during which subjects resumed their normal diet. Both intervention phases were preceded by a 7-day wash-out period. During the wash-out periods and the interventions period the consumption of plants from the order *Brassicales* was not allowed. During each of the intervention the subjects consumed prepatation of *verum* (containing active myrosinase) or control (containing no active myrosinase) every morning and afternoon. Before the first intake of nasturtium (*verum* or control) on day 1 and 14, the participants collect spot urine. Before the first intake and 4 h after the last intake, blood was taken.

A monocentric, randomized, double-blind, controlled, crossover trial was conducted at the Medical Center of the University of Freiburg, Germany (for study design see Figure 2). The intervention was conducted between April and September 2019. Each subject participated in two 14-day intervention phases separated by a 14-day phase-out period during which subjects resumed their normal diet. Both intervention phases were preceded by a 7-day wash-out period. During the wash-out period and the intervention period, subjects were advised to not consume plants from the order *Brassicales*. A list of foods to avoid was provided. During each of the interventions the subjects consumed one serving of *verum* or control every morning and afternoon (with 12 h interval), optionally together with some white bread. The order of consumption (begin with *verum* or control) was assigned randomly using Microsoft® Excel using the RANDBETWEEN function 1;2. “1” was defined as “control” und “2” as “*verum*.” No certain block size was used and the randomization was done for each subject during enrollment by one unblinded investigator. The full intervention protocol can be viewed at the DRKS (DRKS00016548).

### 2.5. Collection and preparation of blood and urine samples

On day one, subjects arrived fasting at the study center. They were asked to provide a spot urine sample, which was immediately placed on ice. Then, before intake of the first preparation, the initial blood samples were drawn into Li-Heparin vacutainers and serum vacutainers with coagulant (Sarstedt AG & Co. KG, Nümbrecht, Germany). Vacutainers for serum samples were placed on ice; Li-Heparin vacutainers for PBMC isolation were kept at room temperature. All samples were processed within 2 h after sampling. On day 14, 4 h after the last procedure in the morning, spot urine and blood were collected as on the first day. On day 14, no further intake took place in the evening. This schedule was chosen because the concentration of ITC metabolites in human serum reaches a maximum between 1 and 5 h after consumption (15).

Urine samples were stored in urine monovette (Sarstedt, Sarstedt AG & Co. KG, Nümbrecht, Germany) and reaction tubes at −80°C until analysis. Two 200 μL urine aliquots were mixed with 50 μL TFA for chemical analysis and stored at −80°C. Blood with coagulant was centrifuged at 4°C and 2000× *g* for 10 min to obtain serum, aliquoted in 200 or 300 μL portions, and stored at −80°C. One 300 μL aliquot was mixed with 100 μL TFA and stored at −80°C until chemical analysis.

### 2.6. Isolation and cultivation of human PBMC

PBMC were isolated from Li-heparinized blood by centrifugation on a LymphoPrep™ gradient using SepMate™ centrifugation tubes (Stemcell™ Technologies Germany GmbH, Cologne, Germany). Cells were washed twice with PBS, and cell viability as well as cell concentration were determined using the trypan blue exclusion test. Cell suspensions of 1 × 10^6^cells/mL were prepared with RPMI, supplemented with 10% FCS, 1% Pen/Strep and glutamine. 1.5 mL of cell suspension was stimulated with 100 ng/mL LPS and incubated at 37°C in a humidified incubator with 5% CO_2_/95% air atmosphere for 3, 24, or 48 h. Subsequently, the cells were centrifuged at 500× *g* for 5 min at 4°C. Cell free supernatants were collected, and stored at −80°C. Cell pellets were washed twice with ice cold PBS and stored at −80°C.

### 2.7. Quantification of PGE_2_ and LTB_4_ release by ELISA assay

Cell free supernatants and serum samples were used for quantification of PGE_2_ and LTB_4_ using the ELISA kits from Cayman (Hamburg, Germany) according to the manufacturer’s instructions.

### 2.8. Quantification of IL-10, IL-1 β, IL-12, and TNF-α by ELISA assay

The quantification of cytokines was done using the human IL-10, IL-1β, IL12, and TNF-α uncoated ELISA kits from Invitrogen (Carlsbad, California, USA) following the manufacturer’s protocol.

### 2.9. Analysis of glucosinolates and their hydrolysis products in nasturtium

Nasturtium plant powder was analyzed for GLS and the potential to release GLS hydrolysis products. For analysis of the GLS hydrolysis products, 1.5 g of the plant powder was prepared as described in 2.3. After 20 s, 500 μL of the mixture were placed into a solvent resistant extraction vessel, which was placed on an ice-water bath. Then, samples were extracted at 0°C and analyzed by GC–MS as described previously (16), except that an Agilent J&W GC VF-5 ms column (30 m × 0.25 mm × 0.25 μM) linked to a 10 m EZ-Guard P/N:CP9013 guard column (both Agilent Technologies, Waldbronn, Germany) was used for separation and the transfer line was set to 270°C. GLS were analyzed from the dry powder in their desulfo-form as described before (17). Three aliquots of active and inactivated plant powder material were analyzed (*n* = 3) for GLS and hydrolysis product formation.

### 2.10. Preparation of samples for determination of mercapturic acid metabolites

Urine and serum samples were prepared for analysis as described before (18). Frozen samples were thawed, vortexed for 1 min and centrifuged at 20,854× *g*, 4°C for 10 min. The supernatant was transferred to SPE cartridges. Prior to transferring, the SPE cartridges were conditioned with 3 mL acetonitrile (ACN) and equilibrated with 3 mL water. After the application of the samples, cartridges were washed with 3 mL formic acid (FA, 0.1% in water) and the analytes were eluted with 3 mL of FA (0.1% in ACN:water, 90:10, v/v). Afterwards, the eluates were evaporated to dryness with a steady stream of nitrogen. The urine and serum samples were reconstituted in 200 μL and 100 μL FA (0.1% in ACN:water, 90:10, v/v), respectively. Aliquots of the samples (5 μL) were then injected into the LC-ESI-MS/MS system.

### 2.11. LC-ESI-MS/MS analysis of the mercapturic acid metabolites

The mercapturic acid metabolites (BITC-glutathione, BITC-GSH; BITC-cysteine, BITC-Cys; BITC-cysteineglycine, BITC-CysGly; BITC-N-acetylcysteine, BITC-NAC) were analysed as described before (15) with minor modifications. The LC-ESI-MS/MS analysis was performed on a 4000 QTrap triple quadrupole MS/MS system (AB Sciex Germany GmbH, Darmstadt, Germany) coupled with an Agilent 1,200 series HPLC system (Agilent Technologies Deutschland GmbH & Co. KG, Waldbronn, Germany). The Software Analyst 1.6.1 (AB Sciex Germany GmbH, Darmstadt, Germany) was used for data acquisition and processing. The separation was performed on a Phenomenex Kinetex C18 column (5 μm, 100 Å, 150 × 2.1 mm). The set temperature for the oven was 20°C and the autosampler temperature was 4°C. The mobile phase consisted of 0.1% FA in water (A) and 0.1% FA in ACN (B) with a constant flow of 300 μL/min. Starting with 80% of A which was held for 1 min before B was raised from 20 to 90% within 11 min and held for 4 min. To equilibrate the system A was raised from 10 to 90% within 1 min and held for 5 min. The MS analysis was done in the negative ionization mode, with an entrance potential of −10 V, a desolvation gas temperature of 450°C, ion spray potential of −4.5 kV, gas 1 and gas 2 at 46 psi and a curtain gas pressure at 10 psi. Quantitation was done by an external calibration curve in a concentration range between 0.01 and 50 μM. The quantity of metabolites in urine was expressed as nmol/mg creatinine.

### 2.12. Preparation of serum samples for determination of protein conjugates

The sample preparation of serum samples was conducted as described before with minor modifications (18). The samples were thawed, vortexed for 1 min and aliquotes of 200 μL were mixed with 500 μL of PBS buffer (pH 7.4). Afterwards, 200 μL of protease solution was added (10 mg/mL in PBS) for protein digestion. The samples were incubated overnight (15 h, 37°C) and the enzyme inactivated by adding 100 μL TFA. After vortexing for 1 min and centrifugation at 20,854× *g*, 4°C for 10 min, the supernatant was applied to prepared SPE cartridges. Preparation of the SPE cartridges was done by conditioning with 3 mL methanol and equilibrating with 3 mL water. After sample application, the cartridges were washed with 3 mL FA (0.1% in water) and the analytes were eluted with 3 mL FA (0.1% in MeOH:water, 90:10, v/v). Subsequently, the serum samples were evaporated to dryness under a gentle stream of nitrogen and redissolved in 100 μL FA (0.1% in MeOH-water, 90:10, v/v). Sample aliquotes of (4 μL) were injected into the LC-ESI-MS/MS system.

### 2.13. LC-ESI-MS/MS analysis of protein conjugates

For the LC-ESI-MS/MS analysis the protocol performed by Kühn and colleagues was used (18). The 4000 QTrap triple quadrupole MS/MS system (AB Sciex Germany GmbH, Darmstadt, Germany) was coupled with an Agilent 1200 series HPLC system (Agilent Technologies Deutschland GmbH & Co. KG, Waldbronn, Germany). Data acquisition and processing was done using the Software Analyst 1.6.1 (AB Sciex Germany GmbH, Darmstadt, Germany). For quantification, an external calibration with BITC-lysine (BITC-Lys) and BITC-Cys synthesized by Kühn et al. (18) was used in a concentration range from 0.01 to 4 μmol/L (18).

### 2.14. Quantification of creatinine

Creatinine was used as a normalization factor to estimate mercapturic acid metabolites in urine. Quantification of creatinine in urine samples was done using the ELISA kit from Invitrogen AG (Carlsbad, CA, USA) following the manufacturer’s protocol.

### 2.15. Statistical study design and analysis

The sample size calculation was based on the primary outcome, namely change in PGE_2_ serum level upon nasturtium intervention. According to this, detection of a 25% difference between groups in PGE_2_ level, measured by ELISA, with 80% power, using a 2-tailed t-test at the 1% significance level and 30% standard deviation, requires 34 participants. The calculation was based on the assumption of a log-normal distribution of the values. Based on an estimated drop-out-rate of 20%, 41 subjects were recruited for the trial. For both intervention phases, the between-subject difference in outcome parameters was calculated. The two-sided Student’s paired t-test or Wilcoxon signed-rank test (due to parameter distribution) was carried out. Normal distribution was assessed using the Kolmogorov–Smirnov test and Shapiro–Wilk test. To analyze for a potential carry-over effect between the *verum* and control intervention phase, a pre-test was performed by calculating the sum of the measured values in the intervention trial for each participant and compared across the two groups by an unpaired t-test (19). This was done for the primary parameter PGE_2_ in serum and LPS stimulated PBMC. Statistical significance was set to *α* ≤ 0.05. Furthermore, an exploratory responder analysis was carried out to see how the intervention affected the individual subject. Data analysis was performed using the statistical software package SAS, version 9.4 (SAS Inc., Cary, NC, USA) or GraphPad Prism 6.0 software (LaJolla, California, USA).

## 3. Results

### 3.1. Study population characteristics and side effects

**Figure 3:**
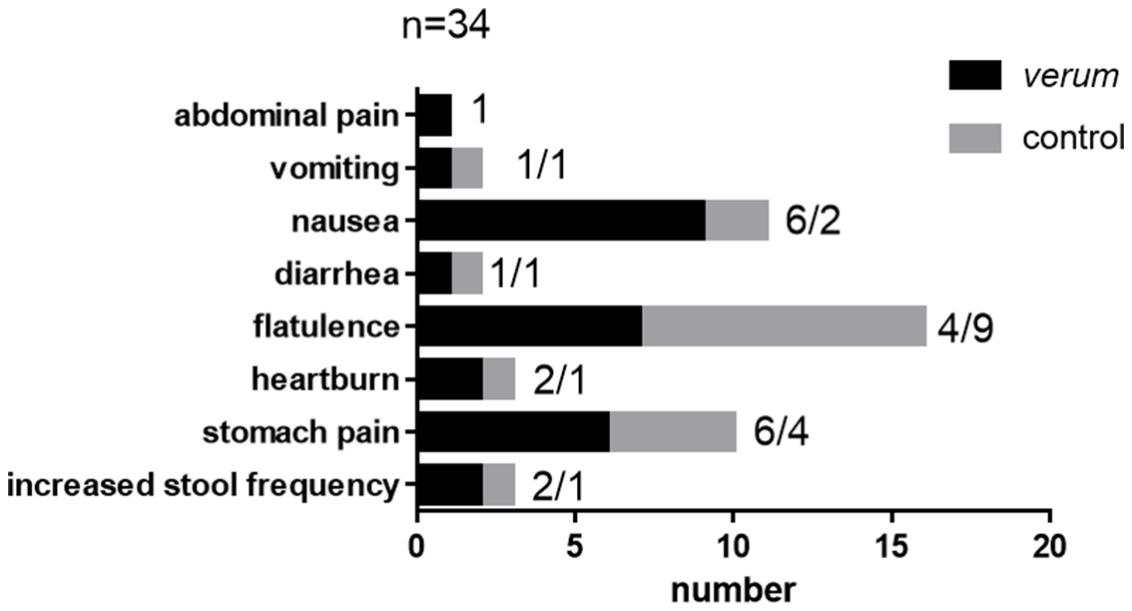
Reported side effects during the intervention with *verum* and control.

A total of 34 healthy female participants completed the intervention trial. Seven individuals dropped out before allocation (Figure 1). Reasons were infection(s) (*n* = 6) and conflicting time schedules (*n* = 1). Individuals were aged 23.32 ± 2.56 years, with a body weight of 60.98 ± 5.19 kg, height of 1.69 ± 0.05 m and a BMI of 21.42 ± 1.79 kg/m^2^. Individuals starting with *verum* were aged 23.48 ± 2.62 years, with a body weight of 60.98 ± 6.13 kg, height of 1.69 ± 0.05 m and a BMI of 21.33 ± 1.97 kg/m^2^. Individuals starting with control were aged 23.08 ± 2.37 years, with a body weight of 61.12 ± 3.39 kg, height of 1.68 ± 0.05 m and a BMI of 21.57 ± 1.57 kg/m^2^. Participants reported mainly mild side effects, which are given in Figure 3. As an example, six subjects in the BITC-containing nasturtium intervention group reported nausea, while this was two in the control group (nasturtium without preformed BITC). For flatulence, it was four and nine, respectively.

### 3.2. Traceability of BITC metabolites and BITC adducts in serum and urine

**Figure 4:**
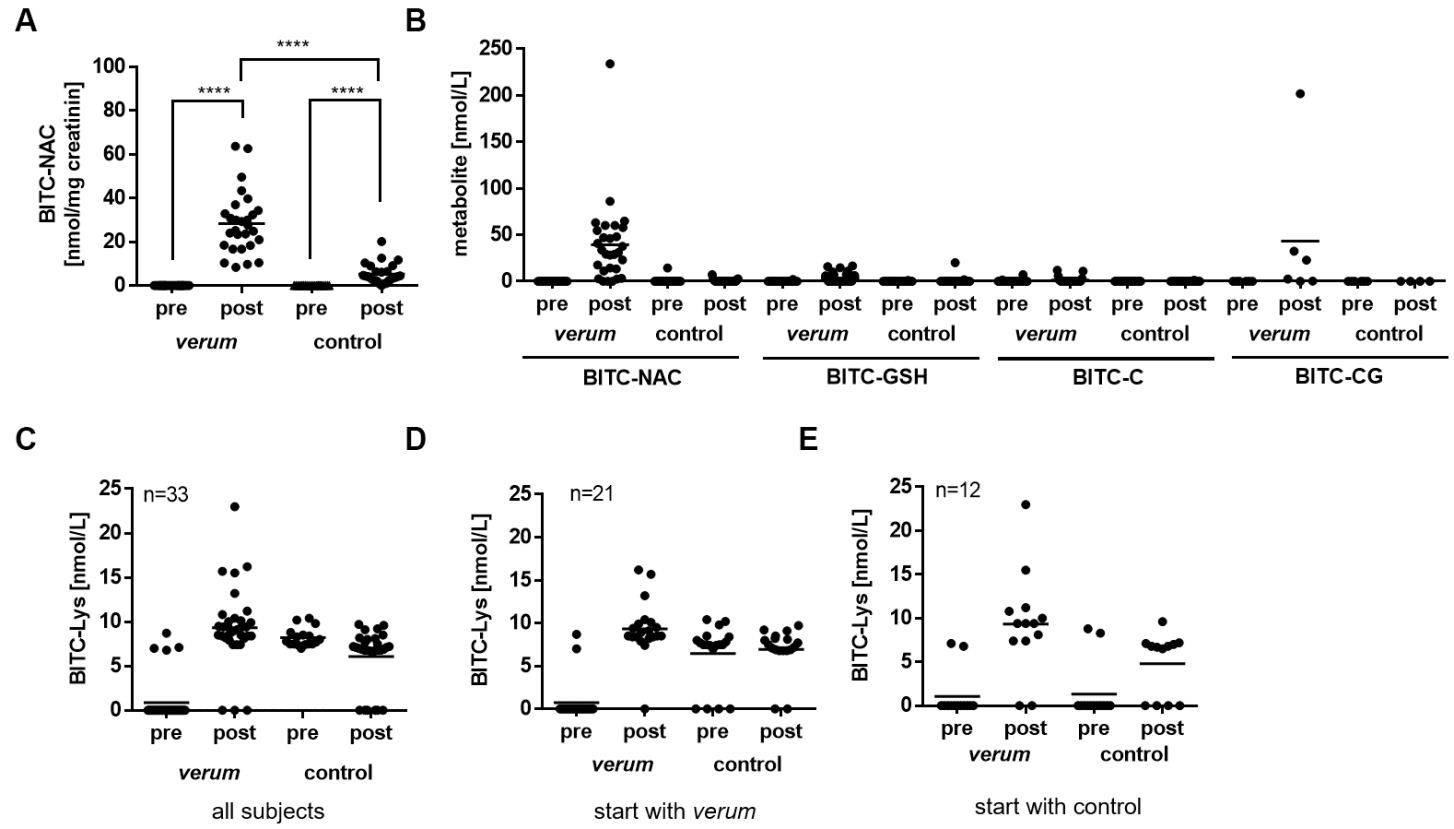
Detection of BITC metabolites and protein conjugates in serum and urine of participants using LC-ESI-MS/MS analysis. BITC-NAC was quantified in urine **(A)** and serum samples **(B)** from subjects before (pre) and after (post) of consumption of *verum* or control. **(C)** BITC-Lys was quantified in serum samples from subjects before (pre) and after (post) from intervention phases *verum* and control. **(D,E)** Concentration of BITC-Lys in serum samples dependent on which preparation was first consumed: *verum* **(D)** or control **(E)**. Each dot represents results from one participant tested and the line represents the mean. Asterisk indicates statistically difference. ****p < 0.0001. BITC, benzyl isothiocyanate; NAC, N-acetylcystein; GSH, glutathione; C, cysteine; CysGly, cysteinylglycine; Lys, lysine.

BITC metabolites from the mercapturic acid pathway were detected in urine and serum samples of subjects before and after both intervention phases. As shown in Figures 4A,B, no BITC metabolites could be detected in all urine and serum samples before intervention, which confirmed the efficacy of the wash-out and compliance of the subjects to the study protocol. BITC-NAC was detected in urine samples of subjects from the *verum* group at a mean level of 28.36 ± 14.12 nmol/mg creatinine, and from the control group at 5.36 ± 4.53 nmol/mg creatinine (Figure 4A). BITC-NAC was detected in serum samples of subjects from *verum* group at a level of 43.91 ± 44.58 nmol/mg, and from control group at 0.58 ± 1.5 nmol/mg (Figure 4B). Additional to BITC-NAC, other metabolites from the mercapturic acid pathway could be detected in serum samples of the *verum* group, albeit at lower levels than BITC-NAC (Figure 4B).

BITC-Lys protein adducts could be detected in serum samples in both intervention groups (*verum* and control, Figure 4C). While in serum samples from only four subjects of the *verum* group BITC-Lys adducts were found before intervention start (mean of 7.4 nmol/L ± 0.88), this was the case in 17 subjects from the control group (mean 7.76 nmol/L ± 2.18), Figure 4C. In contrast to the mercapturic acid metabolites, ITC protein adducts seem to have a relatively long half-life (20). Therefore, it was possible that the measured adducts in the control group resulted from a previous *verum* intervention. We thus re-evaluated these data according to the sequence of the start of the intervention, i.e., *verum* or control (Figures 4D,E) and found that 15 of the 17 positive samples had in fact been preceded by a *verum* intervention.

### 3.3. Influence of nasturtium intervention on COX/PGE_2_ pathway activation

**Figure 5:**
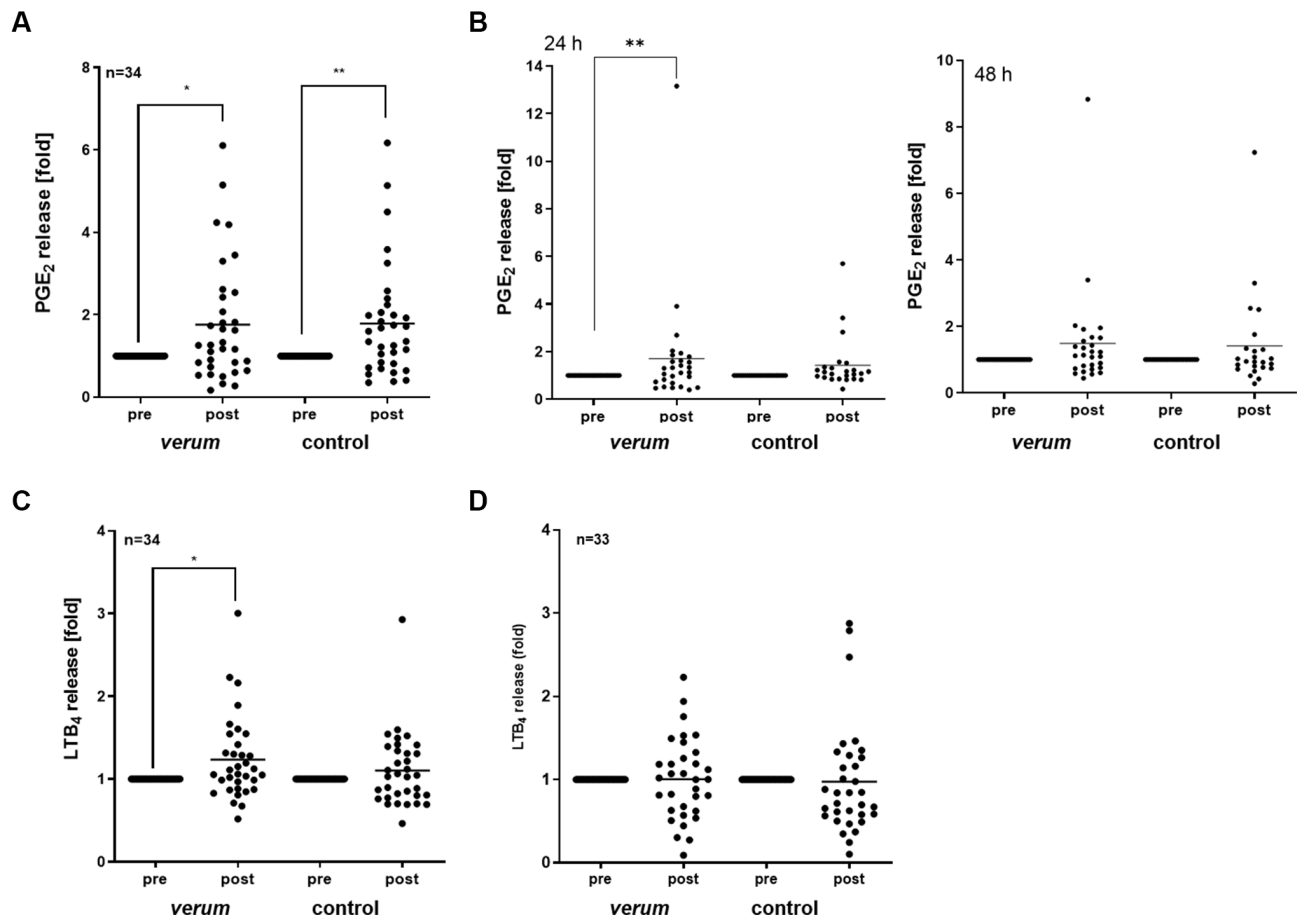
Effects of nasturtium intervention on COX/LOX pathway parameters in serum and LPS stimulated PBMC. PGE_2_ was quantified in serum samples **(A)**. Isolated PBMC were stimulated with 100 ng/mL LPS for 24 or 48 h **(B)**. LTB_4_ was quantified in serum samples **(C)** or 1 h LPS/fMLP stimulated PBMC **(D)**. Data are shown as fold control post/pre. Each dot represents results from one participant tested and the line represents the mean. Asterisk indicates statistically difference. *p < 0.05, **p < 0.01.

First, we analyzed the magnitude of difference in outcome in the *verum* and control group in terms of carrying out a pre to post analysis (Figure 5A). Intervention with *verum* significantly increased the mean PGE_2_ concentration in serum by 1.76-fold (*p* ≤ 0.05). This was comparable to the control (1.78-fold, *p* ≤ 0.01), which indicated an independence of the effect from the BITC content of the plant. Next, we investigated the effect of the intervention on PGE_2_ release in LPS stimulated PBMC in both phases to simulate an acute bacterial infection. Bacterial LPS is commonly used in such experiments to mimic inflammatory responses in cell and animal studies. After intervention, PGE_2_ release from 24 h stimulated PBMC increased to a mean of 1.71-fold (*verum*, p ≤ 0.01), and 1.43-fold (control), see Figure 5B. After 48 h of stimulation, PGE_2_ release was still upregulated upon intervention (*verum*: 1.49-fold, control: 1.43-fold). As seen for the serum PGE_2_ analysis, no significant superior treatment effect of *verum* compared to control was observed.

We additionally analyzed the data for a potential carryover effect between the two intervention phases, but a *p*-value of 0.7498 indicated that this was not the case.

While breakdown of arachidonic acid through the enzymes COX-2 and microsomal prostaglandin E synthase-1 results in PGE_2_ production, it can also lead to LTB4 formation through 5-lipooxygenase (5-LOX) (21). Thus, we also analyzed the effect of intervention with nasturtium on LTB_4_ in both serum as well as LPS/*N*-formylmethionine-leucyl-phenylalanine (fMLP) stimulated PBMC. We observed that the mean LTB_4_ level was significantly increased in serum upon *verum* intervention (1.24-fold, *p* ≤ 0.01, Figure 5C). This was not evident in stimulated PBMC (1.00-fold, *p* = 0.835, Figure 5D).

The response in PGE_2_ to the intervention was quite heterogeneous, although the present study population was similar in many baseline parameters such as age, sex, weight, or height. To determine the effect of the intervention at the individual level, we next categorized subjects with a > 25% decrease in PGE_2_ serum level as “responders,” and those with an increase in PGE_2_ > 25% as “adverse responders.” Following this, 26% of the *verum* group could be classified as “responders,” 52% as “adverse responders,” and 22% showed no relevant change. For the control group, 24% of subjects could be classified as “responders,” 58% as “adverse responders,” and 18% showed no relevant change.

**Figure 6:**
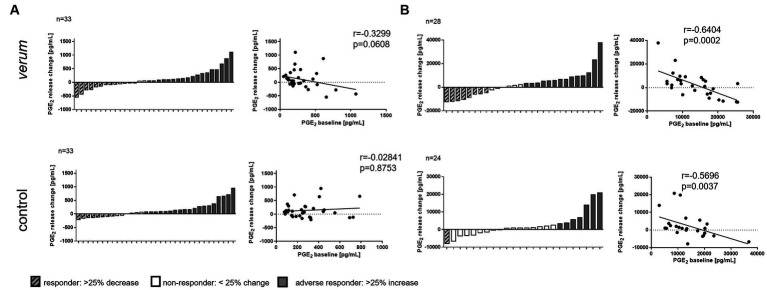
Individual responses to nasturtium intervention. Waterfall diagrams depict the individual change in release of PGE_2_ upon intervention with *verum* or control in serum **(A)** or LPS stimulated PBMC for 24 h **(B)**. Each bar represents one participant. Dot plots show the correlation between baseline PGE2 and change in PGE_2_ level after intervention. Each dot represents one participant. Data for the correlation coefficient (*r*) and significancy (*p*) are given.

Previous studies addressed the question of whether an individual’s outcome could depend on the baseline level of the parameter of interest, e.g., (22). Following this idea, we then first calculated the pre-to-post changes in PGE_2_ levels from each volunteer and correlated these to the corresponding PGE_2_ baseline levels. The results are presented in Figure 6 (A: PGE_2_ serum levels, B: PGE_2_ release from 24 h stimulated PBMC). An inverse correlation could be seen between the baseline PGE_2_ levels and the magnitude of change upon *verum* intervention in serum samples (*verum*: *r* = −0.3299, *p* = 0.0608; control: *r* = −0.02841, *p* = 0.8753), and in stimulated PBMC from both intervention groups (*verum*: *r* = −0.6404, *p* = 0.0002; control: *r* = −0.5696, *p* = 0.003). However, there are some statistical pitfalls in correlating the baseline value with the change in a factor, such as the artifact of regression to the mean or random variation within subjects, as described by Atkinson and Batterham (23). We thus calculated the change and standard deviation (SD) in PGE_2_ levels between the two baselines and compared this with the change upon *verum* or control intervention. In serum samples, the mean change from baseline was 189.5 pg/mL (SD: 345.9, CI: 68.80; 310.2), the change in the *verum* group was 241.9 pg/mL (SD: 252, CI: 154; 329.8), and the change in the control group was 244.3 pg/mL (SD: 311.5, CI: 135.6; 353). Since the mean SD in the baseline change was larger than the mean values in the intervention groups, a regression artifact cannot be excluded. In 24 h stimulated PBMC the mean change from baseline was 5496 pg/mL (SD: 4891, CI: 3381; 7611), the change in the *verum* group was 7862 pg/mL (SD: 7637 CI: 4900; 10,923) and the change in the control group was 4725 pg/mL (SD: 5725, CI: 2308; 7142). In this case, a pseudo effect is unlikely. However, having a relatively small sample size and no comparator arm without any treatment, the validity of this analysis is limited.

### 3.4. Influence of nasturtium intervention on TNF-α and interleukins

**Figure 7:**
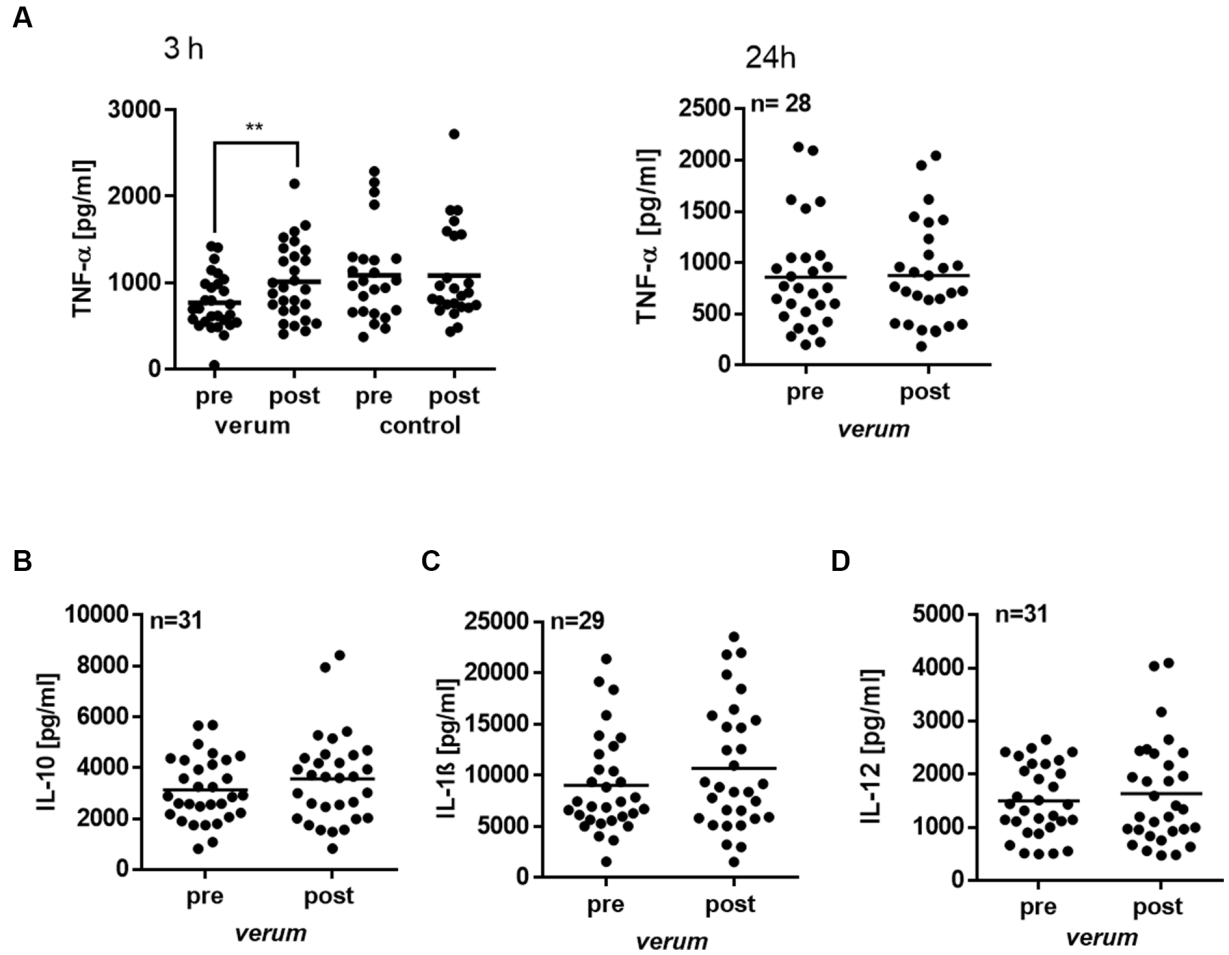
Effect of nasturtium intervention on cytokine release in LPS stimulated PBMC. Isolated PBMC were stimulated with 100 ng/mL LPS for 3 or 24 h before (pre) or after (post) intervention. TNF-α **(A)**, IL-10 **(B)**, IL-1β **(C)** and IL-12 **(D)** cytokines were analyzed by ELISA. Each dot represents the result from one participant tested in duplicates, and the line represents the mean. Asterisk indicates statistically difference. **p < 0.01.

Upon invasion of foreign pathogens such as LPS-generating gram-negative bacteria, an inflammatory cascade is immediately activated. It produces, besides lipid mediators, pro-inflammatory cytokines, such as TNF-α, or IL-1β. As a significant upregulation in PGE_2_ was seen in intervention groups, we also studied the release of cytokines from LPS-activated cells. As given in Figure 7A, intervention with *verum* significantly increased TNF-α release in LPS stimulated PBMC after 3 h (1.65-fold, *p* = 0.0032; pre: mean 780.8 ± 316.4 pg/mL vs. post: mean 1019.62 ± 452.3 pg/mL). Intervention with control *preparation* had no effect on LPS-stimulated PBMC (1.22-fold, *p* = 0.7818; pre: mean 1051 ± 524.2 pg/mL vs. post: mean 1108 ± 503.4 pg/mL). After 24 h, the TNF-α level was back to baseline. No change was seen in the anti-inflammatory cytokine IL-10, or the pro-inflammatory cytokines IL-1β, and IL-12 (Figures 7B–D).

## 4. Discussion

Increased consumption of ITC-containing foods such as nasturtium may improve human health to a certain extent (24). However, randomized, controlled human trials are currently lacking to provide a solid basis for recommending targeted consumption of these foods to protect health.

PGE_2_ is a multifunctional molecule that orchestrates a wide array of biological responses, homeostasis and inflammation in the human body. The ability of PGE_2_ to either activate or suppress biological processes, including inflammation, is context-dependent which reflects the complexity of this receptor system (25). It is involved in temperature regulation (11), crucial for muscle regeneration, or osteoblast bone formation (26, 27). In the early phases of a bacterial infection, PGE_2_ and related prostanoids act as vasodilators. Tissue influx of neutrophils, macrophages, and mast cells from the bloodstream is thereby facilitated, resulting in swelling and edema at the site of infection or tissue injury (28). Furthermore, PGE_2_ controls a number of processes that result in the clearance of the inflammation and subsequent tissue repair (25, 29). Using LPS-activated PBMC from humans, we found *in vitro* that aqueous nasturtium extract concentration-dependently decreased PGE_2_ levels at concentration corresponding to BITC 0.15 to 4.2 nmol/mL (8), suggesting that nasturtium might suppress an acute inflammatory response upon bacterial infection. A recent human crossover intervention trial (*n* = 19) with single intake of fresh watercress (*Nasturtium officinale*) reported a mild pro-inflammatory reaction in LPS-stimulated whole blood cultures from healthy subjects upon consumption of watercress as compared to control group, though. This was shown by significantly increased mean levels of TNF-α, IL-1β, and IL-6 (30). In the present study, we found that mean PGE_2_ serum levels increased upon nasturtium intervention as compared to baseline. In LPS stimulated PBMC, this was seen for PGE_2_, as well as for TNF-α. In the intervention group, we detected low level of BITC-NAC (43.91 ± 44.58 nmol/L) in serum after the *verum* intervention. Compared to a recent *in vitro* study, BITC concentration achieved in serum was very low. This could also be an explanation for the different effect on PGE_2_. However, we observed considerable variation in response between individuals, with some subjects showing an increase in PGE_2_ response while in another part it was decreased upon intervention. This concurs with other studies, in which usually only up to 40% of subjects responded to intervention, e.g., (22). Reasons may include individual factors, like genetic differences or factors such as the composition of the gut microbiome (31). Further analysis of the present data indicated that individuals with relatively high baseline PGE_2_ levels may be more likely to respond with suppression of the prostanoid to nasturtium intake and vice versa. However, the hypothesis of such adaptogenic properties of the plant needs further validation, because these additional analyses were only of exploratory nature.

In the present study, it was investigated whether BITC significantly accounts for the biological effects of nasturtium. Thus, nasturtium with inactivated myrosinase as control was used. The absence of BITC formation during nasturtium preparation could be confirmed by chemical analysis. No relevant BITC metabolites were detected in serum samples of the subjects after intake of the control preparation. In urine, the level of BITC in the control group was only 19% of that found in the *verum* group, which concurs with a low, unavoidable GLS conversion by intestinal bacteria (32). Consequently, present results here suggest that BITC does not play a crucial role for the observed effect of nasturtium on PGE_2_.

While baseline serum and urine samples confirmed the absence of BITC before intervention, we found BITC-Lys protein conjugates in a number of the serum samples. ITC protein adducts are considered to be more stable biomarkers reflecting a larger time span of ITC exposure history. Stable albumin adducts have a reported half-life of 20–25 days (20, 33). In the present intervention study, we reprocessed these data according to the respective onset of the first round of intervention. This revealed that BITC-Lys adducts were already mainly present in the baseline sample of the control group that had previously received the *verum* intervention. Thus, it can be assumed that the time between the two intervention phases was not sufficient to eliminate all ITCs. The extent to which these remaining adducts may have influenced the results reported here is impossible to say. Protein adducts have been discovered to be involved in the chemopreventive effects of ITCs (34). Statistical data analysis gave, however, no clear evidence for a relevant carry-over effect between intervention groups. To ensure that contamination does not occur between study phases, we recommend that future experiments consider even greater separation of intervention phases.

In contrast to the previously reported *in vitro* results, on average LPS activated PBMC from *verum* or control group showed increased PGE_2_ levels. This was also true for subjects’ serum samples, indicating that the results were independent from the BITC content of nasturtium. Using the mean values, individual differences are ignored though, and we observed a high variability in individual responses as reaction to the treatment. The responder analysis performed here suggests that PGE_2_ formation after intervention depends whether or not baseline PGE2 was high, but this needs to be further verified in larger studies. Such a relationship could help predict the effectiveness of nasturtium for modulating PGE_2_ on the level of the individual in the future. It also provides clues for the subsequent design and analysis of studies on this medicinal plant, which may allow us to better determine clinically significant effects in patient outcomes. In the long term, identifying phenotypes that respond differently to an intervention may thus improve the delivery of personalized nutrition or phytopharmaceutical drugs and optimize the outcome for the individual.

## Data availability statement

The original contributions presented in the study are included in the article/supplementary material, further inquiries can be directed to the corresponding author.

## Ethics statement

The studies involving human participants were reviewed and approved by the Ethical Committee of the University of Freiburg (ethical vote number 322/18, 11/13/2018). This study was conducted in accordance with the Declaration of Helsinki. This intervention trial was registered on German Clinical Trials Register (DRKS) with the ID: DRKS00016548. The patients/participants provided their written informed consent to participate in this study.

## Author contributions

EL: conceptualization. LF, HT, JS, MK, and FH: methodology. CH, LF, HT, SC, JS, MK, FH, MA, NB, and EL: formal analysis. CH, LF, HT, SC, JS, MK, and FH: investigation. RS, MS, and EL: resources. CH, LF, and EL: writing—original draft preparation. FH, SR, and MS: writing—review and editing. All authors have read and agreed to the published version of the manuscript. The listing order of the authors is without weighting.

## Funding

The intervention trial was partly supported by a grant from Repha GmbH, Langenhagen, Germany. Repha GmbH was not involved in the design, conduction, interpretation, or publishing of the results. FH was funded by the Leibniz Association (J16/2017). The manuscript processing fee was partly funded by the University of Freiburg in the funding program Open Access Publishing.

## Conflict of interest

The authors declare that the research was conducted in the absence of any commercial or financial relationships that could be construed as a potential conflict of interest.

## Publisher’s note

All claims expressed in this article are solely those of the authors and do not necessarily represent those of their affiliated organizations, or those of the publisher, the editors and the reviewers. Any product that may be evaluated in this article, or claim that may be made by its manufacturer, is not guaranteed or endorsed by the publisher.
